# Association between Inflammation-Based Prognostic Markers and Mortality in Patients Admitted to Intensive Care Units

**DOI:** 10.3390/diagnostics14161709

**Published:** 2024-08-06

**Authors:** Ah Ran Oh, Jeong-Am Ryu, Seung Joo Lee, Chung Su Kim, Sangmin Maria Lee

**Affiliations:** 1 Department of Anesthesiology and Pain Medicine, Samsung Medical Center, Sungkyunkwan University School of Medicine, Seoul 06351, Republic of Korea; 2 Department of Critical Care Medicine, Samsung Medical Center, Sungkyunkwan University School of Medicine, Seoul 06351, Republic of Korea; 3 Department of Neurosurgery, Samsung Medical Center, Sungkyunkwan University School of Medicine, Seoul 06351, Republic of Korea

**Keywords:** albumin, biomarker, C-reactive protein, Intensive care unit, mortality, SOFA

## Abstract

Background: We compared the prognostic value of the C-reactive protein (CRP)-to-albumin ratio (CAR), neutrophil-to-lymphocyte ratio (NLR), and modified Glasgow prognostic score (mGPS) with the Sequential Organ Failure Assessment (SOFA) score in an intensive care unit (ICUs). Methods: This study used the data of 53,877 adult patients admitted to an ICU between June 2013 and May 2022. Using the CAR, NLR, and mGPS values, as well as the SOFA score from the ICU, we conducted multivariable logistic regression analysis and used the receiver operating characteristic (ROC) curve to compare the predictive value for 28-day and 1-year mortality. Results: A total of 2419 patients (4.5%) died within 28 days, and 6209 (11.5%) patients died within 1 year. After an adjustment, all predictors were found to be independent risk factors for 28-day mortality (odds ratio [OR] 1.31, 95% confidence interval [CI] 1.29–1.33, *p* < 0.001 for the SOFA score; OR 1.05, 95% CI 1.03–1.07, *p* < 0.001 for CAR; OR 1.01, 95% CI 1.00–1.02, *p* < 0.001 for the NLR; and OR 1.19, 95% CI 1.08–1.30, *p* < 0.001 for the mGPS). This trend persisted for the 1-year mortality. In ROC curve analysis, the CAR showed better predictability than the NLR and mGPS. Furthermore, the predictive power of the CAR was significantly higher than that of the SOFA score for 1-year mortality. Conclusions: The CAR, NLR, and mGPS values at ICU admission were independent risk factors of mortality after ICU admission. The predictive value of CAR was higher than that of the SOFA score for 1-year mortality. CAR assessment at ICU admission may be a feasible predictor of long-term mortality.

## 1. Introduction

The assessment of prognosis in patients admitted to intensive care units (ICUs) is a major concern in critical care due to the high rates of morbidity and mortality [1,2]. Different scoring systems have been established to predict patient prognosis and develop treatment plans. The Sequential Organ Failure Assessment (SOFA) score is an effective and well-validated system for measuring the severity of acute illness and predicting clinical outcomes in critically ill patients [3,4]. The strength of the SOFA scoring system is a comprehensive reflection of various organic systems [5], but it requires detailed clinical data, which places limits on the prompt evaluation of rapidly changing conditions [6]. Considering the importance of early recognition of the risks for adverse outcomes in ICU treatment, deploying simpler scoring systems at ICU admission could be useful for the quicker initiation of appropriate management.

A number of simplified markers based on inflammation have recently been found to be useful parameters for predicting the prognosis of critically ill patients. These include biological markers such as the C-reactive protein (CRP)-to-albumin ratio (CAR), neutrophil-to-lymphocyte ratio (NLR), and modified Glasgow prognostic score (mGPS). These markers have been shown to be closely related to the degree of inflammation and have been used as independent predictors of mortality in ICU patients [7,8,9,10,11]. In addition, these markers are easy and cost-effective to measure, which allows the prompt evaluation of a patient’s status and subsequent risk of mortality. However, the value of these inflammatory markers has not been evaluated in comparison with that of the SOFA score, which evaluates the actual function of vital organs. Therefore, the present study aimed to compare the predictive value of the aforementioned inflammatory markers (the CAR, NLR, and mGPS) and the SOFA score in predicting 28-day and 1-year mortality in patients admitted to an ICU.

## 2. Methods

This retrospective study was approved by the institutional review board of Samsung Medical Center (SMC 2022-07-050). Our registry was generated in deidentified form, so the requirement for written informed consent was waived. We conducted this study following the Declaration of Helsinki and report the results according to the Strengthening the Reporting of Observational Studies in Epidemiology guidelines.

### 2.1. Data Curation and Study Population

This registry included 65,654 consecutive adult patients who were admitted to the ICU at Samsung Medical Center, Seoul, Republic of Korea, between June 2013 and May 2022, for whom the SOFA scores at ICU admission were recorded. To generate this large single-center cohort, we used an institutional electronic archive system that provides the data of >4 million patients with >900 million laboratory findings and >200 million prescriptions. Specifically, we used the “Clinical Data Warehouse Darwin-C” electronic system to search and retrieve medical records in deidentified form. To update mortality data outside our institution, this system uses a unique identification number assigned to each patient from the National Population Registry of the Korea National Statistical Office. After raw medical records were extracted, independent investigators blinded to the mortality outcome organized the relevant variables. Blood laboratory test results and SOFA scores were automatically extracted, and we excluded those patients without available laboratory test results at the time of ICU admission from this study.

### 2.2. Definitions and Study Endpoints

The SOFA score evaluates the performance of six different organ systems, including the respiratory, cardiovascular, hepatic, coagulation, renal, and neurological systems [12,13]. The SOFA score is routinely measured at admission to an ICU and was included for analysis. The SOFA score was calculated with the worst value recorded during the initial 24 h after ICU admission. The CAR was defined as the ratio of CRP (mg/L) to albumin (g/dL), and the NLR was defined as the ratio of absolute neutrophil count to the lymphocyte count. The mGPS value was also determined based on the levels of CRP and albumin as follows: (1) mGPS 2 = CRP > 10 mg/L and albumin < 3.5 g/dL, (2) mGPS 1 = CRP > 10 mg/L and albumin ≥ 3.5 g/dL, and (3) mGPS 0 = CRP ≤ 10 mg/L with or without hypoalbuminemia [14]. For subgroup analysis, ICUs were grouped into five types: medical, cardiac, surgical, oncologic, and neurologic. The primary endpoint was 28-day mortality after ICU admission, while the secondary endpoint was 1-year mortality after ICU admission.

### 2.3. Statistical Analysis

Baseline characteristics are presented as mean ± standard deviation values for continuous variables and numbers with percentages for categorical variables. To evaluate each prognosticator as a risk factor for mortality, we performed univariable and multivariable logistic regression analyses. In the multivariable logistic regression analysis, a stepwise backward elimination method was applied using the variables with *p* < 0.05 in the univariable analysis. The results are presented as odds ratios (ORs) with 95% confidence interval (CI) values.

We assessed the predictability of values of the CAR, NLR, and mGPS and the SOFA score for mortality via receiver operating characteristic (ROC) curve analysis for both the entire study population and patients admitted to each type of ICU. Because the CAR showed the highest predictive power among the biological markers we assessed, the area under the ROC curve (AUC) of the CAR was compared to that of the SOFA score using DeLong’s test [15]. All statistical analyses in this study were performed with R version 4.2.0 (R Foundation for Statistical Computing, Vienna, Austria; http://www.r-project.org/, accessed on 19 July 2024), and *p* < 0.05 was considered to be statistically significant.

## 3. Results

**Figure 1:**
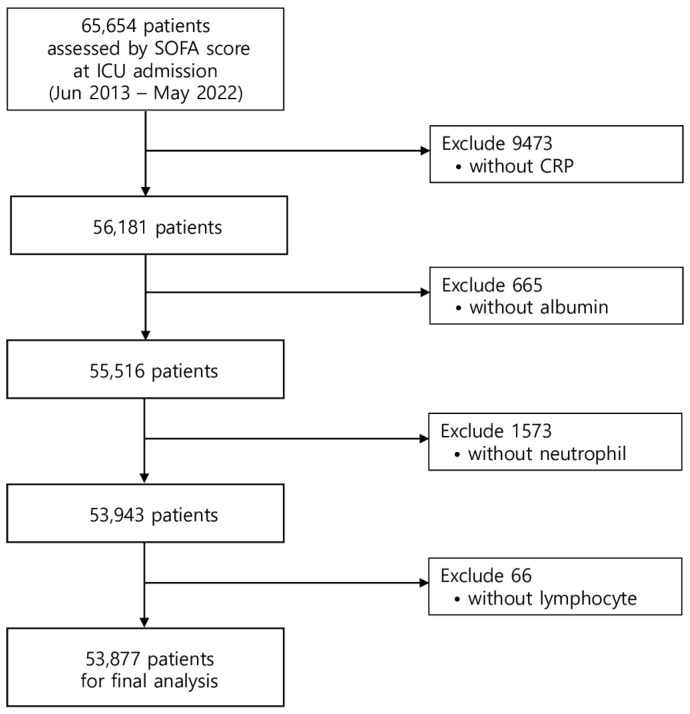
Patient selection flowchart.

From the entire registry of 65,654 patients with SOFA scores at ICU admission who were assessed in this study, we excluded 11,777 patients without available blood CRP, albumin, neutrophil, and/or lymphocyte values at ICU admission (Figure 1). A total of 53,877 (82.1%) patients were enrolled for final analysis. During the follow-up period, 2419 patients (4.5%) died within 28 days, and 6209 (11.5%) patients died within 1 year after ICU admission. The baseline characteristics of the patients according to their mortality outcome are summarized in Table 1.

The results of the logistic regression analyses for 28-day and 1-year mortality are shown in Table 2. The multivariable logistic regression analysis showed that all predictors were independent risk factors of 28-day mortality (OR 1.31, 95% CI 1.29–1.33, *p* < 0.001 for the SOFA score; OR 1.05, 95% CI 1.03–1.07, *p* < 0.001 for the CAR; OR 1.01, 95% CI 1.00–1.02, *p* < 0.001 for the NLR; and OR 1.19, 95% CI 1.08–1.30, *p* < 0.001 for the mGPS). This trend was also observed for the 1-year mortality (OR 1.16, 95% CI 1.15–1.17, *p* < 0.001 for the SOFA score; OR 1.14, 95% CI 1.12–1.16, *p* < 0.001 for the CAR; OR 1.00, 95% CI 1.00–1.00, *p* = 0.01 for the NLR; OR 1.08, 95% CI 1.00–1.16, *p* = 0.04 for the mGPS). In the subgroup analysis according to ICU type, we determined that the NLR was not significantly associated with the 28-day mortality in the surgical ICU (OR 1.00, 95% CI 0.99–1.01, *p* = 0.08) (Table 3).

**Figure 2:**
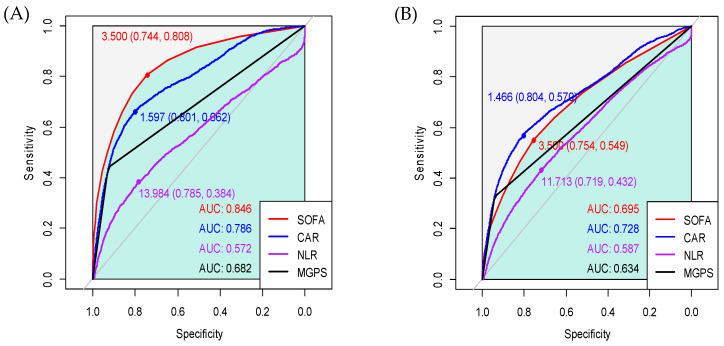
Receiver operating characteristic curves of the CAR, NLR, and mGPS values and the SOFA score for (**A**) 28-day mortality and (**B**) 1-year mortality after ICU admission.

**Figure 3:**
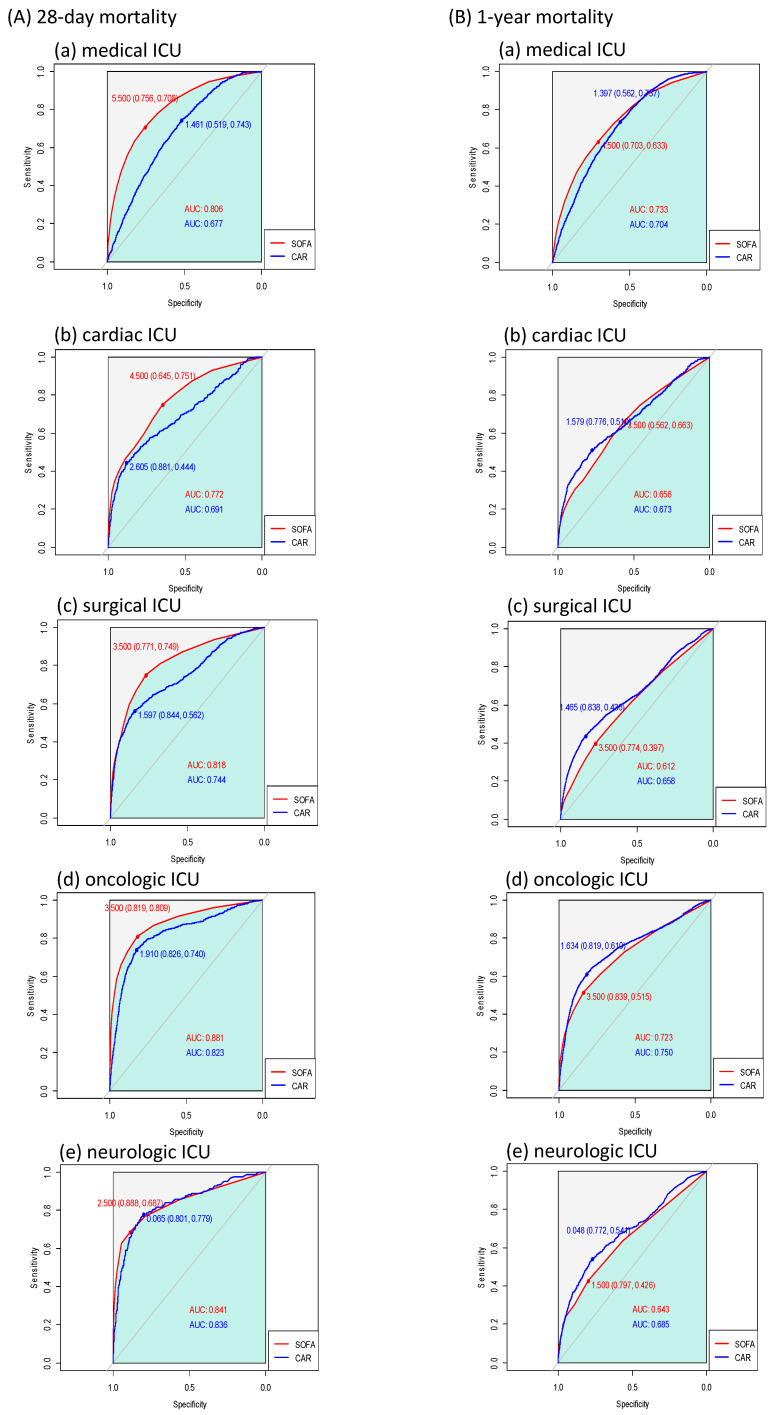
Receiver operating characteristic curves of CAR and SOFA score according to ICU type for (**A**) 28-day mortality and (**B**) 1-year mortality.

The ROC curves of each variable for 28-day and 1-year mortality are presented in Figure 2. From the analysis of the prediction of 28-day mortality, the AUCs of the SOFA score, CAR, NLR, and mGPS were 0.846, 0.786, 0.572, and 0.682, respectively. The AUC of the SOFA score was significantly higher than that of the CAR (0.846 vs. 0.786, Z = 9.43, *p* < 0.001). From the analysis of the prediction of 1-year mortality, the AUC of the SOFA score was significantly lower than that of the CAR (0.695 vs. 0.728, Z = −7.46, *p* < 0.001). There were similar trends in the ROC curves for each type of ICU patient, but the SOFA score outperformed the CAR in the prediction of mortality regardless of follow-up period in medical ICU patients (Figure 3). Additionally, the correlation matrix showed that the Pearson correlation coefficients with age were 0.080 for the SOFA score, 0.098 for the CAR, −0.038 for the NLR, and 0.063 for the mGPS. There were no strong correlations between age and any of these prognostic markers. The correlation matrix revealed varying relationships between the different reasons for ICU admission and the prognostic scales (the SOFA, CAR, NLR, and mGPS). Cardiovascular disease and respiratory distress showed positive correlations with the SOFA, CAR, and mGPS, indicating higher scores for these conditions. Neurological disorders and perioperative management negatively correlated with these scales, suggesting lower scores. Post-cardiac-arrest syndrome correlated positively with the SOFA, while severe trauma had very weak positive correlations across all scales. 

The multivariable adjustment included the SOFA, C-reactive protein/albumin ratio, neutrophil/lymphocyte ratio, modified Glasgow prognostic score, being male, malignancy, diabetes mellitus, chronic kidney disease, chronic liver disease, chronic obstructive pulmonary disease, stroke, heart failure, smoking, alcoholic use, and causes of ICU admission.

## 4. Discussion

In this study, we investigated the clinical usefulness of inflammation-based prognostic markers measured at ICU admission for the prediction of mortality and compared their predictive abilities with that of the SOFA score. All inflammatory markers were found to be independent predictors of mortality at 28 days and 1 year after ICU admission, but only the CAR achieved a fair degree of predictability. For the 1-year mortality rate, the predictive power of the CAR was significantly higher than that of the SOFA score, suggesting that the CAR may be a feasible and convenient marker for predicting the mortality of ICU patients, especially during long-term follow-up, compared to the SOFA score, a traditional prognostic factor.

In critically ill patients, the prediction of prognosis is important for the monitoring of the treatment response and the allocation of limited resources [16]. The SOFA score is one of the most validated systems for predicting the prognosis of ICU patients and has the advantage of being relatively simple compared to other scoring systems. Nevertheless, the SOFA score still requires the measurement of numerous variables, which cannot be achieved depending on the treatment provided. In contrast, inflammation-based markers are easily accessible and have been shown to be good predictors of morbidity and mortality in various critical settings. This study evaluated the prognostic value of CAR, NLR, and mGPS values and their efficacy by comparing them to a well-known conventional scoring system (the SOFA score) in critically ill patients. These markers were chosen for our analysis because they are easily measurable using routine laboratory tests and have been shown to correlate strongly with inflammation and nutritional status, both of which are critical factors in patient prognosis. While other indices such as the monocyte-to-lymphocyte ratio (MLR), platelet-to-lymphocyte ratio (PLR), systemic immune-inflammation index (SIRI), albumin–globulin ratio (AGR), and aggregate inflammatory index (AII) also have potential prognostic value, we focused on the CAR, NLR, and mGPS due to their established use in clinical practice and previous studies demonstrating their relevance in critical care settings [7,8,9,10,11]. Our results revealed the best predictive value was achieved with CAR, which was superior to the SOFA score for predicting long-term mortality.

The superiority of the predictive value of the CAR over the SOFA score differed according to the duration of follow-up. The results of our analysis over 28 days of follow-up were similar to previously reported results, where major conventional scoring systems tended to have better prognostic value than the CAR [7,17]. Considering that the acute progression of multiorgan failure is a major cause of early death after ICU admission [18], it is reasonable that the SOFA score performs better in predicting short-term mortality by evaluating the vital organs more systematically. Additionally, the assessment of long-term prognosis in critical care is also essential for more personalized and extended treatment planning [19]. The prediction of long-term prognosis can be more complicated because it involves more complex interactions among the patient’s baseline status and the severity of inflammation [20,21]. In this regard, the CAR outperformed the SOFA score for the 1 year of follow-up by assessing patients’ inflammatory and nutritional status simultaneously.

We also conducted a subgroup analysis in relation to the 28-day mortality according to the type of ICU patient. The SOFA score, CAR, and mGPS values were found to be risk factors for 28-day mortality across all ICU types. This finding shows that the combination of CRP and albumin may be widely used to predict mortality among critically ill patients regardless of ICU type. In contrast, the NLR was not found to be significantly associated with 28-day mortality in the surgical ICU, which emphasizes that nutritional status may need to be considered along with inflammatory status in surgical patients. However, the association between the NLR and mortality might be attenuated by the heterogeneity in surgery type, as numerous studies have documented a significant association in more homogenous populations [22,23,24]. Therefore, further investigation is required to confirm our results.

Several limitations should be considered when interpreting our results. First, this was a retrospective single-center study, so our findings may not be generalizable to different populations. Additionally, known confounding factors were well controlled using multivariable analysis, but the observed associations could still have been affected by residual confounding factors. Second, we excluded patients without SOFA scores or certain laboratory test results at ICU admission, which may have resulted in selection bias due to the inclusion of more complicated patients who were expected to require more intensive treatment. Third, we did not report causes of death, which made it hard to establish a direct relationship between inflammatory status at ICU admission and mortality. Also, whether inflammatory markers are modifiable risk factors in ICU patients was not elucidated in this study. Lastly, blood cultures were performed for a limited number of patients, which may impact the interpretation of our results regarding the potential interference of bloodstream infections with inflammatory markers. Nevertheless, our study has significant value in that it is the first study to demonstrate a relationship between inflammatory markers at ICU admission and mortality and to compare their prognostic values with that of the SOFA score in various ICU patients.

## 5. Conclusions

In this study, the CAR, NLR, and mGPS values and the SOFA score at ICU admission were independent risk factors of 28-day and 1-year mortality after ICU admission. Especially, the predictive value of the CAR was higher than that of the SOFA score for 1-year mortality. Therefore, the CAR at ICU admission may be a feasible predictor of long-term mortality in critically ill patients. Further prospective studies with larger cohorts are necessary to corroborate our findings.

## Figures and Tables

**Table 1:** Baseline characteristics of the patients with mortality.

	Entire Population (*N =* 53,877)	28-Day Mortality (*N =* 2419)	One-Year Mortality (*N =* 6209)
SOFA score at ICU admission	2 (0–4)	7 (4–11)	4 (1–8)
C-reactive protein/albumin ratio	0.49 (0.06–1.39)	3.04 (0.91–6.74)	1.94 (0.45–5.07)
Neutrophil/lymphocyte ratio	7.90 (4.33–12.82)	14.33 (4.76–34.09)	10.09 (5.42–17.82)
Modified Glasgow prognostic score			
0	48,547 (90.1)	1345 (55.6)	4134 (66.6)
1	672 (1.2)	62 (2.6)	122 (2.0)
2	4658 (8.6)	1012 (41.8)	1953 (31.5)
C-reactive protein, mg/dL	1.72 (0.21–4.67)	8.42 (2.69–17.59)	5.72 (1.35–13.40)
Albumin, g/dL	3.5 (3.0–3.9)	2.8 (2.4–3.2)	2.9 (2.5–3.3)
Neutrophil, %	83.0 (74.5–87.7)	83.2 (72.0–89.6)	84.0 (76.0–89.0)
Lymphocyte, %	10.5 (6.7–17.3)	8.0 (4.4–15.0)	8.2 (5.0–14.0)
Age	60.47 (±13.84)	63.11 (±14.08)	63.35 (±14.42)
Male	31,879 (59.2)	1537 (63.5)	4037 (65.0)
Comorbidities			
Hypertension	22,095 (59.2)	1010 (41.8)	2524 (40.7)
Malignancy	21,118 (39.2)	537 (22.2)	2239 (36.1)
Diabetes mellitus	11,941 (22.2)	651 (26.9)	1644 (26.5)
Chronic kidney disease	2350 (4.4)	140 (5.8)	322 (5.2)
Chronic liver disease	3041 (5.6)	269 (11.1)	600 (9.7)
Chronic obstructive pulmonary disease	1960 (3.6)	108 (4.5)	267 (4.3)
Stroke	2604 (4.8)	107 (4.4)	283 (4.6)
Heart failure	837 (1.6)	61 (2.5)	132 (2.1)
Coronary artery disease	3448 (6.4)	170 (7.0)	413 (6.7)
Habitual risk factors			
Current smoker	4647 (8.6)	190 (7.9)	443 (7.1)
Alcohol intake	10,223 (19.0)	364 (15.0)	781 (12.6)
Cause of ICU admission			
Severe trauma	149 (0.3)	12 (0.5)	18 (0.3)
Perioperative management	40,481 (75.1)	391 (16.2)	249 (40.1)
Post-cardiac-arrest syndrome	312 (0.6)	118 (4.9)	143 (2.3)
Neurological disorder	3050 (5.7)	135 (5.6)	298 (4.8)
Respiratory distress	3243 (6.0)	829 (34.3)	1460 (23.5)
Cardiovascular disease	3831 (7.1)	404 (16.7)	799 (12.9)
Abdominal disorder	892 (1.7)	173 (7.2)	305 (4.9)
Others	1919 (3.6)	357 (14.8)	695 (11.2)
ICU management			
Mechanical ventilation	10,699 (19.9)	1449 (59.9)	2641 (42.5)
Continuous renal replacement therapy	1496 (2.8)	505 (20.9)	719 (11.6)
Extracorporeal membrane oxygenation	1405 (2.6)	171 (7.1)	284 (4.6)
Use of vasopressor	8282 (15.4)	1109 (45.8)	1874 (30.2)

Data are presented as *n* (%) or mean (±standard deviation) or median (interquartile). Abbreviations: SOFA, Sequential Organ Failure Assessment; OR, odds ratio; ICU, intensive care unit.

**Table 2:** Baseline characteristics and risk factors of mortality.

	No Mortality	Mortality	Unadjusted Analysis		Adjusted Analysis	
**28-day Follow-up**	***N =* 51,458**	***N =* 2419**	**OR (95% CI)**	***p*-value**	**OR (95% CI)**	***p*-value**
SOFA score at ICU admission	1 (0–4)	7 (4–11)	1.41 (1.40–1.43)	<0.001	1.31 (1.29–1.33)	<0.001
C-reactive protein/albumin ratio	0.45 (0.06–1.28)	3.04 (0.91–6.74)	1.32 (1.30–1.33)	<0.001	1.05 (1.03–1.07)	<0.001
Neutrophil/lymphocyte ratio	7.84 (4.33–12.83)	14.33 (4.76–34.09)	1.00 (1.00–1.01)	<0.001	1.01 (1.00–1.02)	<0.001
Modified Glasgow prognostic score			3.12 (2.99–3.26)	<0.001	1.19 (1.08–1.30)	<0.001
0	47,202 (91.7)	1345 (55.6)				
1	610 (1.2)	62 (2.6)				
2	3646 (7.1)	1012 (41.8)				
**One-year Follow-up**	***N =* 47,668**	***N =* 6209**	**OR (95% CI)**	***p*-value**	**OR (95% CI)**	***p*-value**
SOFA score at ICU admission	1 (0–3)	4 (1–8)	1.24 (1.23–1.25)	<0.001	1.16 (1.15–1.17)	<0.001
C-reactive protein/albumin ratio	0.42 (0.05–1.17)	1.94 (0.45–5.07)	1.33 (1.32–1.35)	<0.001	1.14 (1.12–1.16)	<0.001
Neutrophil/lymphocyte ratio	7.71 (4.22–12.57)	10.09 (5.42–17.82)	1.00 (1.00–1.00)	<0.001	1.00 (1.00–1.00)	0.01
Modified Glasgow prognostic score			2.78 (2.69–2.87)	<0.001	1.08 (1.00–1.16)	0.04
0	44,413 (93.2)	4134 (66.6)				
1	550 (1.2)	122 (2.0)				
2	2705 (5.7)	1953 (31.5)				

Data are presented as *n* (%) or mean (±standard deviation) or median (interquartile). Abbreviations: SOFA, Sequential Organ Failure Assessment; OR, odds ratio; ICU, intensive care unit.

**Table 3:** Risk factors of mortality during 28 days of follow-up according to type of ICU.

	No Mortality	Mortality	Unadjusted Analysis	
**Medical ICU**	***N =* 6887**	***N =* 1533**	**OR (95% CI)**	***p*-value**
SOFA score at ICU admission	3 (1–5)	8 (5–12)	1.33 (1.32–1.35)	<0.001
C-reactive protein/albumin ratio	1.34 (0.17–4.19)	3.75 (1.41–7.45)	1.13 (1.12–1.15)	<0.001
Neutrophil/lymphocyte ratio	7.72 (3.76–14.86)	10.16 (4.00–21.50)	1.01 (1.01–1.02)	<0.001
Modified Glasgow prognostic score			1.61 (1.52–1.71)	<0.001
0	4788 (69.5)	744 (48.5)		
1	256 (3.7)	43 (2.8)		
2	1843 (26.8)	746 (48.7)		
**Cardiac ICU**	***N =* 12,125**	***N =* 330**		
SOFA score at ICU admission	3 (1–6)	8 (4–12)	1.33 (1.29–1.37)	<0.001
C-reactive protein/albumin ratio	0.64 (0.20–1.47)	1.94 (0.52–4.63)	1.35 (1.31–1.39)	<0.001
Neutrophil/lymphocyte ratio	6.38 (3.49–11.77)	10.01 (5.69–17.50)	1.03 (1.03–1.04)	<0.001
Modified Glasgow prognostic score			2.76 (2.44–3.13)	<0.001
0	4788 (69.5)	744 (48.5)		
1	256 (3.7)	43 (2.8)		
2	1843 (26.8)	746 (48.7)		
**Surgical ICU**	***N =* 42,092**	***N =* 805**		
SOFA score at ICU admission	1 (0–3)	7 (3–10)	1.39 (1.36–1.41)	<0.001
C-reactive protein/albumin ratio	0.41 (0.05–1.09)	2.00 (0.41–5.21)	1.37 (1.34–1.39)	<0.001
Neutrophil/lymphocyte ratio	7.97 (4.56–12.71)	10.00 (5.00–17.48)	1.00 (0.99–1.01)	0.08
Modified Glasgow prognostic score			3.42 (3.16–3.69)	<0.001
0	40,167 (95.4)	537 (66.7)		
1	307 (0.7)	16 (2.0)		
2	1618 (3.8)	252 (31.3)		
**Oncologic ICU**	***N =* 22,992**	***N =* 1097**		
SOFA score at ICU admission	1 (0–3)	7 (4–11)	1.59 (1.56–1.63)	<0.001
C-reactive protein/albumin ratio	0.68 (0.25–1.45)	4.07 (1.82–7.66)	1.34 (1.32–1.36)	<0.001
Neutrophil/lymphocyte ratio	8.78 (5.73–13.00)	8.89 (2.95–20.44)	1.01 (1.00–1.01)	<0.001
Modified Glasgow prognostic score			3.54 (3.32–3.78)	<0.001
0	20,976 (91.2)	518 (47.2)		
1	220 (1.0)	23 (2.1)		
2	1796 (7.8)	556 (50.7)		
**Neurologic ICU**	***N =* 9179**	***N =* 163**		
SOFA score at ICU admission	0 (0–1)	4 (2–7)	1.89 (1.78–2.01)	<0.001
C-reactive protein/albumin ratio	0.02 (0.01–0.05)	0.40 (0.08–1.68)	1.66 (1.54–1.80)	<0.001
Neutrophil/lymphocyte ratio	5.25 (2.35–9.95)	11.33 (6.35–18.01)	1.06 (1.05–1.08)	<0.001
Modified Glasgow prognostic score			5.58 (4.29–7.17)	<0.001
0	9110 (99.2)	137 (84.0)		
1	18 (0.2)	3 (1.8)		
2	51 (0.6)	23 (14.1)		

Data are presented as *n* (%) or mean (±standard deviation) or median (interquartile). Abbreviations: SOFA, Sequential Organ Failure Assessment; OR, odds ratio; ICU, intensive care unit.

## Data Availability

Available on reasonable request from the corresponding author.

## Abbreviations

ICU, intensive care unit; SOFA, Sequential Organ Failure Assessment; CRP, C-reactive protein; CAR, C-reactive protein-to-albumin ratio; NLR, neutrophil-to-lymphocyte ratio; mGPS, modified Glasgow prognostic score; ROC, receiver operating characteristic; AUC, area under the receiver operating characteristic curve.
